# Design of Highly Selective Gas Sensors via Physicochemical Modification of Oxide Nanowires: Overview

**DOI:** 10.3390/s16091531

**Published:** 2016-09-20

**Authors:** Hyung-Sik Woo, Chan Woong Na, Jong-Heun Lee

**Affiliations:** Department of Materials Science and Engineering, Korea University, Seoul 02841, Korea; greyshades@korea.ac.kr (H.-S.W.); cwna@korea.ac.kr (C.W.N.)

**Keywords:** nanowires, gas sensors, selectivity, surface modification, CVD

## Abstract

Strategies for the enhancement of gas sensing properties, and specifically the improvement of gas selectivity of metal oxide semiconductor nanowire (NW) networks grown by chemical vapor deposition and thermal evaporation, are reviewed. Highly crystalline NWs grown by vapor-phase routes have various advantages, and thus have been applied in the field of gas sensors over the years. In particular, *n*-type NWs such as SnO_2_, ZnO, and In_2_O_3_ are widely studied because of their simple synthetic preparation and high gas response. However, due to their usually high responses to C_2_H_5_OH and NO_2_, the selective detection of other harmful and toxic gases using oxide NWs remains a challenging issue. Various strategies—such as doping/loading of noble metals, decorating/doping of catalytic metal oxides, and the formation of core–shell structures—have been explored to enhance gas selectivity and sensitivity, and are discussed herein. Additional methods such as the transformation of *n*-type into *p*-type NWs and the formation of catalyst-doped hierarchical structures by branch growth have also proven to be promising for the enhancement of gas selectivity. Accordingly, the physicochemical modification of oxide NWs via various methods provides new strategies to achieve the selective detection of a specific gas, and after further investigations, this approach could pave a new way in the field of NW-based semiconductor-type gas sensors.

## 1. Introduction

Single crystalline metal oxide semiconductor nanowires (NWs) grown by vapor phase reaction have been widely used in various fields because of their large surface-to-volume ratio, excellent thermal stability, and low tendency to form aggregates [1,2]. Among their numerous applications such as photodetectors, photocatalysts, dye-sensitized solar cells, light emitting diodes (LED), etc., the use of semiconductor metal oxide NWs as gas sensors has been extensively studied over the past few decades [3,4,5,6,7,8,9,10]. The porous network of NWs allows rapid diffusion of analyte gases to the entire surface of the NWs, enabling high gas response and short response time. The porous network structure is also advantageous to the uniform doping and loading of catalytic materials on the sensor surface. Moreover, the high crystallinity of the NWs provides thermal stability of sensors [11] and the gas response can be enhanced or controlled by tuning the diameter of the NWs and the NW-to-NW contact configuration [12]. To date, wide-band gap *n*-type semiconductors such as SnO_2_ [13], ZnO [14], In_2_O_3_ [15], and WO_3_ [16] have been prepared in the form of NW networks to explore their potential for gas sensor applications. The growth of *n*-type oxide semiconductor NWs and their application as gas sensors have been reviewed by several authors [3,4,7,17]. Although literature studies on gas sensors using *n*-type metal oxide NWs report high gas response, good stability, and fast response/recovery kinetics, the control of selectivity to gases other than highly reactive C_2_H_5_OH and NO_2_ is still at a nascent stage, limiting the widespread use of oxide NWs for detecting various harmful, toxic, and environmental gases. The main difficulty in achieving selective gas detection emanates primarily from a simple gas-sensing mechanism involving charge transfer by the reaction between analyte gas and adsorbed negatively charged oxygen or the adsorption of analyte gas. Availability of diverse sensing materials or hetero-nanostructures with different gas-sensing characteristics would be more advantageous to design highly selective gas sensors. Thus, oxide NWs with different compositions as well as physicochemical modification of oxide NWs are necessary. Recently, through active research, various groups have reported synthetic routes to decorate metal oxide nanoclusters on the surface of oxide NWs in order to obtain catalytic promotion and control the near-surface space-charge layer. The successful transformation of *n*-type into *p*-type NWs via cation exchange reaction has added to the number of possible combinations of materials. Moreover, various other methods have been employed to achieve selective gas detection in NW-based gas sensors, which include the doping of catalytic materials, the formation of core-shell nanostructures, and the preparation of branched hierarchical structures.

In this overview, early and recent literature strategies aimed to enhance the gas selectivity of oxide NW-based gas sensors via surface modification and hetero-nanostructure design will be reviewed. The main focus of this review will be directed at the achievement of gas selectivity using oxide NW-based gas sensors for practical applications and the comprehension of the underlying gas-sensing mechanism.

## 2. Metal Oxide Nanowires

### 2.1. Growth of Single Crystalline Metal Oxide Nanowires

**Figure 1:**
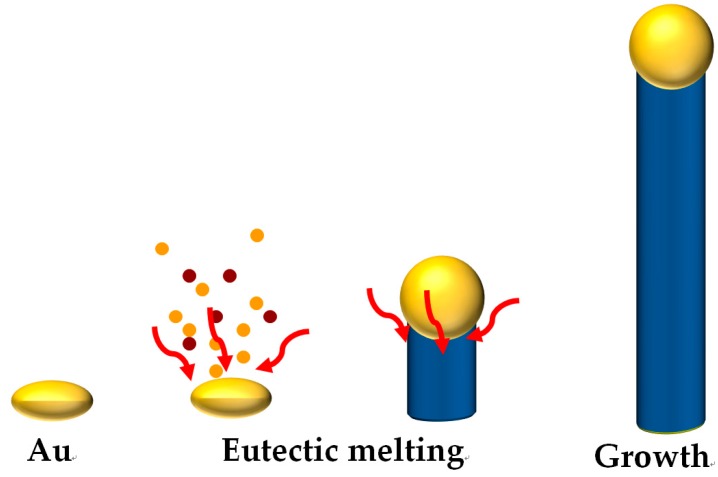
Schematic illustration of the vapor-liquid-solid mechanism.

Metal oxide NWs can be prepared by either a solution-based hydrothermal route or vapor-phase reactions such as chemical vapor deposition (CVD) and thermal oxidation. Compared to the hydrothermal reaction, the vapor-phase methods are more advantageous to prepare single crystalline oxide NWs with a high length-to-diameter ratio. In general, vapor-phase synthetic routes result in the growth of NWs by the vapor-liquid-solid (VLS) mechanism. The VLS mechanism is the most renowned process to grow highly crystalline oxide NWs; it was first introduced by Wagner et al. [18] in the 1960s and has been practiced over the years by many research groups. The VLS process begins with the formation of nanosized liquid droplets of growth catalyst (usually Au) at elevated temperatures on the surface of a substrate (usually Si). The gaseous source reactants adsorb on the liquid metal followed by nucleation. The continuous dissolution of the reactant in the liquid droplet results in the supersaturation of the source material at the liquid-solid interface, which leads to the growth of single crystalline NWs (Figure 1). Such NWs can grow to be several tens of nanometers in diameter and up to several tens of micrometers in length. Not only binary metal oxide NWs such as SnO_2_, ZnO, and WO_3_ [13,14,19], but also ternary metal oxide NWs such as Zn_2_SnO_4_ [20] and ITO (indium tin oxide) [21], are prepared by VLS mechanism. The thermal oxidation process of metal foil can also be used to prepare single crystalline metal oxide NWs. Iron, copper, vanadium, niobium, and zinc foil have been explored for the preparation of single crystalline metal oxide NWs [22,23,24,25,26]. NW network structures can be fabricated by the thermal oxidation of patterned metal (Cu and Zn) thin film with a micrometer-scale gap [27,28,29]. However, since oxide NWs are grown on continuous metal foils with aligned structures, they usually do not form networked structures. Furthermore, the lengths of NWs grown by oxidation are generally shorter than those of NWs grown by the CVD method.

### 2.2. Gas-Sensing Mechanism of Metal Oxide Nanowire Networks

Single crystalline NWs grown via thermal evaporation method show a highly gas-accessible and porous interwoven network morphology that is an advantageous feature in the gas-sensing reaction. The sensor signal of such NW network gas sensors is due to the change in resistance of NWs between two or more electrodes in air and the presence of analyte gas. The sensor resistance either increases or decreases when exposed to reducing gases, depending on the type of majority carrier in the metal oxide semiconductor NWs. In the case of *n*-type NWs such as SnO_2_, ZnO, and In_2_O_3_, oxygen molecules in air adsorb on the surface of the NWs and take electrons from the NWs, becoming negatively charged (O_2_^−^, O^−^, or O^2−^). This causes the formation of an electron depletion layer near the surface due to the decrease in electron density, which leads to high resistance in air. When a reducing gas is present, gas molecules react with the adsorbed oxygen on the NW surface and return the electrons to the metal oxide NW, causing a decrease of resistance. The species of adsorbed negatively charged oxygen (O_2_^−^, O^−^, or O^2−^) change according to sensor temperature [30,31], which determine the dependence of gas response on gas concentration [32,33]. Clearly, the full electron depletion can be achieved when the diameter of the NWs becomes comparable or smaller than twice the electron depletion layer thickness [34]. Thus, ultrathin NWs [35] and hierarchical nanostructures [36] assembled from thin nanorods are beneficial to enhance the gas response. In the case of nanoparticles, a size reduction often results in the agglomeration of particles into larger secondary particles by van der Waals attraction. The gas response of large and dense agglomerates is low [37,38,39] because the inner part of secondary particles is inactive to the sensing reaction. In contrast, crystalline NWs have a lower tendency to form agglomerates owing to their networked structure. Thus, all of the NWs participate in the gas-sensing reaction. Additionally, the numerous NW-to-NW contacts also contribute to the enhancement of the gas-sensing reaction since the potential junctions at the contact points constitute a chemiresistive region. Therefore, an increase in the number density of NWs in the network will result in an increase in gas response as well as sensor resistance [12,40].

### 2.3. Metal Oxide Nanowire Network Gas Sensors: Strengths and Limitations

In general, single crystalline metal oxide NWs show various advantages for gas sensors such as high reproducibility, thermal stability, high surface-to-volume ratio, and low tendency to form agglomerates. However, there are also limitations to the application of oxide NWs in the field of gas sensors. To date, studies on gas sensors using metal oxide NWs are mainly focusing on a few limited *n*-type metal oxides such as SnO_2_, ZnO, In_2_O_3_, and WO_3_ for their ease in NW growth. Although these oxide NWs generally show high responses and selectivity to C_2_H_5_OH and NO_2_ [13,14,15,16], the research on the selective detection of other gases remains at a nascent stage. However, from the viewpoint of practical applications, many different gases should be detected in a selective and sensitive manner. For example, various indoor air pollutants such as benzene, xylene, and toluene exist at low concentrations and are difficult to detect using the above *n*-type oxide semiconductor NWs due to their relatively low reactivity. Moreover, the selective detection of low-concentration biomarker gases such as H_2_S, acetone, NO, NH_3_, and CO from exhaled breath is highly required to diagnose halitosis, diabetes, asthma, renal failure, and bronchiectasis, respectively [41,42,43,44,45,46,47]. This suggests that various new compositions of oxide NWs, catalytic materials, and hetero-nanostructures should be considered to achieve diverse applications of gas sensors.

## 3. Physicochemical Modifications for the Enhancement of Selectivity

Since the gas-sensing reaction of metal oxide semiconductor NW network gas sensors occurs on the surface, the reduction of the diameter and increase of the surface-to-volume ratio are beneficial to achieve high gas response. If the diameter of the *n*-type oxide semiconductor NW is smaller than twice the electron depletion layer, also known as the Debye length, the NW can be regarded as “fully depleted”, in which case, the resistance variation upon exposure to reducing gases is drastically enhanced [48,49]. However, the synthesis of ultrathin NWs with a diameter below ~20 nm is difficult when using the conventional thermal evaporation method; further, even when achieved, this approach enhances the gas response, while still leaving the selective detection of a specific gas a challenging problem. Therefore, additional strategies have been investigated over the years to further enhance the selectivity of gas sensors using metal oxide NW networks.

### 3.1. Noble Metal Doping/Loading

The loading of noble metal catalysts has been intensively studied in gas sensors using oxide nanoparticles [50,51,52,53]. Accordingly, in the initial stages of the research on gas sensors using oxide NWs, various noble metals such as Pt, Pd, Au, and Ag have been loaded to explore the possibility of enhancing the gas-sensing characteristics. Representative literature results are summarized in Table 1 [54,55,56,57,58,59,60,61,62,63,64,65,66,67,68]. Noble metals are primarily known to enhance the gas response either by “electronic sensitization” or “chemical sensitization”. “Electronic sensitization” is the enhancement of the gas response by tuning the charge carrier concentration, while “chemical sensitization” promotes the reaction between adsorbed oxygen and spilled over analyte gas from the catalytic material causing an increase of gas response [69]. Ag and Pd are the most representative electronic sensitizers that exhibit a change of oxidation state in air and reducing gas (AgO and PdO in air, and metal in reducing gas), while Pt is the most well-known chemical sensitizer to promote the spillover effect [48].

Aside from the gas response enhancement, the doping and loading of noble metal catalysts also increases the selectivity of NW network-based gas sensors. For example, Au is known to facilitate CO oxidation even at low temperatures because of the low binding energy for CO adsorption on Au (111) surfaces [70,71,72]. Further, Pt [55,73,74] and Pd [62] are known to enhance selectivity to H_2_, while Ag reportedly increases selectivity to C_2_H_5_OH [66,67,75,76].

Many research groups have used noble metals for either doping or loading on metal oxide NWs in order to achieve selective gas detection. In general, when loading noble metals on the surface of NWs, ultrafine nanoparticles with narrow size distributions should be dispersed uniformly. Under this configuration, the electron depletion layer formed beneath the catalyst particles can overlap with each other, enhancing the effect of electronic sensitization, while the spillover effect on the catalyst surface via chemical sensitization can also be maximized.

In NW-based gas sensors, the loading of Pt is known to enhance the selectivity of SnO_2_ NW sensors to NO_2_, toluene, and H_2_ [54,55,56], while Pt-loaded ZnO-based NW gas sensors showed selective detection of C_2_H_5_OH [57]. The loading of Pt enhanced the response of In_2_O_3_ NW sensors to O_2_ and H_2_ [58,59]. However, the role of noble metal catalysts is not always consistent, probably due to the complex nature of the surface reaction at various sensing temperatures and the different interaction between sensing and catalytic materials. Moreover, the gas responses to several different gases often increase together with the catalyst loading, which may limit the tuning of gas selectivity.

### 3.2. Transition Metal Oxide Doping/Decoration

To achieve the selective detection of different gases, in addition to the use of noble metal catalysts, distinct catalytic materials should be employed in the design of gas sensors. Instead of expensive noble metals, transition metal oxides such as Co_3_O_4_, Mn_3_O_4_, Cr_2_O_3_, NiO, and CuO with high catalytic activity to volatile organic compounds can be considered as a viable alternative to promote the gas-sensing reaction [77]. Over the past few years, many research groups have studied and reported the doping or decorating of various transition metals in/on metal oxide NWs for the enhancement of selectivity. In addition, other catalytic materials such as the oxides of Mo, Mg, and Sb have been also explored to enhance gas selectivity. Data from the literature are summarized in Table 2 [78,79,80,81,82,83,84,85,86,87,88,89,90].

**Figure 2:**
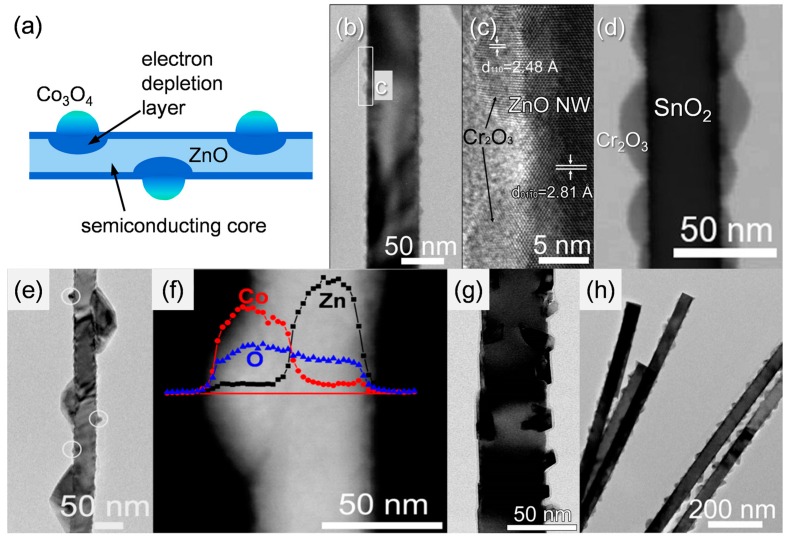
(**a**) Schematic diagram of the electron depletion layer in *n*-type oxide semiconductor nanowires decorated with *p*-type oxide semiconductor nanoclusters; TEM images of (**b**,**c**) Cr_2_O_3_-decorated ZnO nanowires; (**d**) Cr_2_O_3_-decorated SnO_2_ nanowires; (**e**,**f**) Co_3_O_4_-decorated ZnO nanowires; (**g**) NiO-decorated ZnO nanowires; and (**h**) Mn_3_O_4_-decorated ZnO nanowires. Reproduced from [80,81,82,83,84] with permission; (**a**,**e**–**g**) [82,83] Copyright (2011,2012) The Royal Society of Chemistry; (**b**,**c**) [80] Copyright (2012) IOP Publishing Ltd.; (**d**) [81] Copyright (2014) Elsevier; (**h**) [84] Copyright (2012) American Chemical Society.

**Figure 3:**
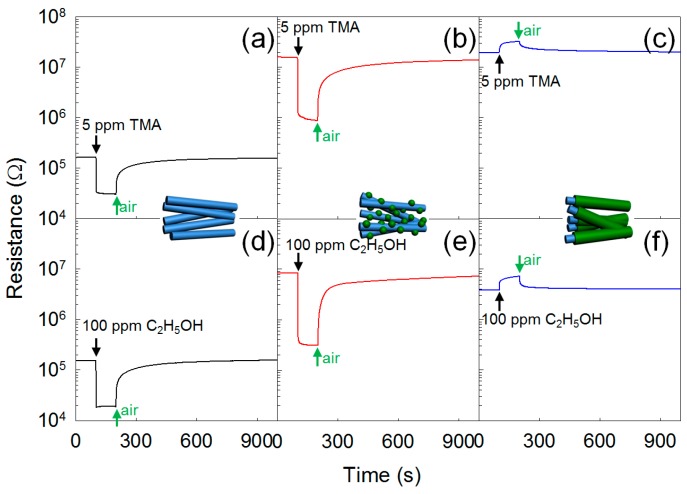
Dynamic sensing transients of (**a**) ZnO NWs; (**b**) Cr_2_O_3_-decorated ZnO NWs; and (**c**) ZnO-Cr_2_O_3_ core-shell nanocalbes (NCs) to 5 ppm trimethylamine (TMA) at 400 °C. Dynamic sensing transients of (**d**) ZnO NWs; (**e**) Mn_3_O_4_-decorated ZnO NWs; and (**f**) ZnO-ZnMn_2_O_4_ core–shell NCs to 100 ppm C_2_H_5_OH at 400 °C. Adapted from [80,84] with permission; (**a**–**c**) [80] Copyright (2012) IOP Publishing Ltd.; (**d**–**f**) [84] Copyright (2012) American Chemical Society.

**Figure 4:**
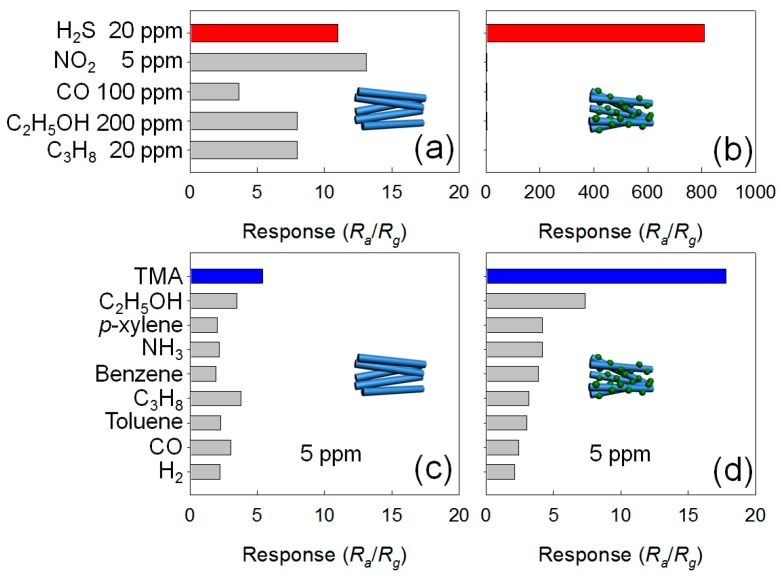
Gas selectivity of (**a**) pristine SnO_2_ NW network sensor and (**b**) CuO-decorated SnO_2_ NW network sensors at 300 °C; gas selectivity of (**c**) pristine ZnO NW network sensor and (**d**) Cr_2_O_3_-decorated ZnO NW network sensor at 400 °C. Adapted from [80,91] with permission; (**a**,**b**) [91] Copyright (2009) Elsevier; (**c**,**d**) [80] Copyright (2012) IOP Publishing Ltd.

It should be noted that transition metal oxides such as Co_3_O_4_, Mn_3_O_4_, Cr_2_O_3_, NiO, and CuO are *p*-type oxide semiconductors. The decoration of discrete *p*-type metal oxide nanoclusters on *n*-type oxide semiconductor NWs provides various pathways to tailor their gas-sensing characteristics. Some examples of *n*-type oxide NWs decorated with different *p*-type oxide semiconductor nanoclusters are shown in Figure 2 [80,81,82,83,84]. Nanoscale *p-n* junctions are formed, which cause the expansion of the electron depletion layer in *n*-type NWs beneath the *p*-type nanoclusters (Figure 2a). This increases the resistance in air (R_a_) and makes the sensor more sensitive to the injection of charges by the gas-sensing reaction, enabling a larger chemiresistive variation (Figure 3a,b,d,e) [80,84]. Thus, the response of oxide NW-based gas sensors can be significantly enhanced without thinning the diameter of the oxide NWs down to twice the thickness of the electron depletion layer. This has a practical meaning because the preparation of oxide NWs thinner than ~20 nm is a relatively difficult task. In addition, the gas selectivity can also be controlled when the decorated materials show high catalytic activity towards a specific gas. For example, the decoration of CuO on SnO_2_ NWs dramatically enhances the selectivity to H_2_S [91] (Figure 4a,b). This can be explained by the transformation of resistive CuO-SnO_2_ *p-n* junctions into highly conducting CuS-SnO_2_ metal-*n* junctions upon exposure to H_2_S. The gas selectivity emanates from the high chemical affinity between CuO and H_2_S. The present authors [80,81] uniformly decorated Cr_2_O_3_ nanoparticles on the surface of CVD-grown ZnO (Figure 2b,c) and SnO_2_ NWs (Figure 2d) using the thermal evaporation of CrCl_2_ powder. The decoration of Cr_2_O_3_ both on ZnO (Figure 4c,d) and SnO_2_ NW sensors (not shown) significantly enhanced the selectivity to trimethylamine (TMA). Cr and Cr_2_O_3_ are known for their unique methylamine selective adsorption and dissociation characteristics through bonding with the nitrogen lone pair electrons [92]. Since TMA has similar structure and chemical properties to methylamine, the response to TMA gas could be greatly improved by decorating the surface of metal oxide NWs with catalytic Cr_2_O_3_, enabling selective detection. By using the same synthetic method with different source powders, Co_3_O_4_-, NiO-, and Mn_3_O_4_-decorated ZnO NWs [82,83,84] (Figure 2e–h) were also synthesized, showing the potential to tune the selectivity to NO_2_ and C_2_H_5_OH.

All the *n*-type oxide NWs decorated with *p*-type oxide semiconductor nanoclusters exhibited *n*-type sensing behavior (decreased resistance upon exposure to reducing gases) (Figure 3b,e), which proves that the *p*-type nanoparticles play the role of catalyst and expand the adjacent electron depletion layer of *n*-type NWs while the conduction is carried out through the backbone *n*-type NWs. Furthermore, the expansion of the electron depletion layer due to the formation of nanoscale *p-n* junctions was proven by the increase of sensor resistance of approximately two orders of magnitude (Figure 3a,b,d,e) upon the decoration of *p*-type clusters on *n*-type oxide NWs [82,83,84]. This method of catalytic material decoration requires fine tuning of source evaporation temperature and reaction time for the formation of nanoparticles with discrete configuration and uniform size distribution. When a uniform decoration of catalytic nanoparticles on the surface of NWs is successfully achieved, both selectivity and sensitivity of the gas sensor can be greatly enhanced by the combination of the catalytic action and nanoscale *p-n* junction formation.

The acid and base properties of sensing/additive materials can also be used to achieve selective gas detection. Alkali and acidic oxides are known to promote dehydrogenation of ethanol and dehydration, respectively [93]. The former reaction is advantageous to enhance the response and selectivity to C_2_H_5_OH. Thus, the addition of an alkali oxide to NWs can be used to enhance ethanol selectivity. For instance, the La_2_O_3_-decorated SnO_2_ NWs reported by Hieu et al. [85] showed enhanced response to C_2_H_5_OH gas due to the basic properties of La_2_O_3_ nanoparticles.

**Figure 5:**
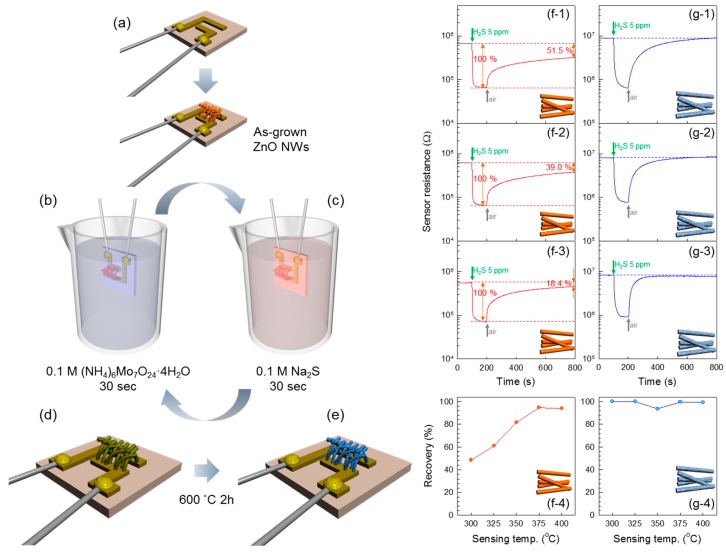
(**a**–**e**) Schematic illustration of the synthesis process of Mo-doped ZnO NW network gas sensors. Sensing transients of pure and Mo-doped ZnO NW network gas sensors to 5 ppm H_2_S at 300, 325, and 350 °C: (**f-1**) ZnO NW sensor, 300 °C; (**f-2**) ZnO NW sensor, 325 °C; (**f-3**) ZnO NW sensor, 350 °C; (**g-1**) Mo-doped ZnO NW sensor, 300 °C; (**g-2**) Mo-doped ZnO NW sensor, 325 °C; and (**g-3**) Mo-doped ZnO NW sensor, 350 °C; (**f-4**,**g-4**) Recovery (%) = (R_air-recovery_ − R_gas-H2S_)/(R_air-fresh_ − R_gas-H2S_) × 100 (%) of pure and Mo-doped ZnO NW sensors at 300–400 °C (where, R_air-fresh_: sensor resistance in air before exposure to H_2_S, R_gas-H2S_: sensor resistance in 5 ppm H_2_S, and R_air-recovery_: sensor resistance in air after 10 min exposure to air). Reproduced from [88] with permission. Copyright (2014) The Royal Society of Chemistry.

The doping of metal ions into *n*-type oxide semiconductor NW sensors can be also used to achieve selective detection. For example, the doping of Mo has been used to increase the selectivity to H_2_S in ZnO NW sensors. Previously, the chemical reaction between H_2_S gas and CuO leading to the formation of a metallic CuS phase was suggested as a strategy to achieve H_2_S selectivity in CuO-loaded *n*-type oxide semiconductor NW sensors [94]. Although the H_2_S selectivity was high, the recovery of gas sensors functionalized with Cu-based catalyst was usually sluggish or, in the worst case, exhibited incomplete recovery kinetics [95,96]. This failure was related to the slow conversion of CuS into CuO during recovery, which triggered the search for a new catalytic material that could detect H_2_S gas not only in a selective but also highly reversible manner. The present authors [88] doped Mo in ZnO NW networks by the successive ionic layer adsorption and reaction (SILAR) method and subsequent heat treatment (Figure 5a–e). It should be noted that Mo and MoO_3_ with high affinity to H_2_S, just like CuO, are advantageous to achieve selective H_2_S sensing. Pure ZnO NW sensors showed incomplete recovery after the H_2_S-sensing event (Figure 5(f-1–f-4)) since a zinc sulfide layer was formed on the surface of ZnO NWs upon exposure to H_2_S (known as “reactive adsorption”). In contrast, Mo-doped ZnO NWs exhibited complete recovery and stable sensing characteristics as well as enhanced gas response to H_2_S throughout a wide range of operating temperatures (Figure 5(g-1–g-4)). MoO_3_ is known to form regenerable adsorbents that do not go through reactive adsorption and conversion to sulfide when exposed to H_2_S [97], which explains the reversible sensing characteristics of Mo-doped ZnO NW sensors. This suggests that not only the chemical affinity between additives and analyte gas but also the adsorption species on additives should be taken into account for selective and reversible sensing characteristics.

The doping of basic materials such as Mg can be used to enhance the gas selectivity in metal oxide NW sensors [89]. Mg-doped ZnO NWs were synthesized by using MgO as catalyst for the VLS growth of ZnO NWs. When Mg is doped in ZnO NWs, it is known to increase the energy band gap of ZnO, resulting in the decrease in charge carrier concentration [98,99,100]. This will induce a larger chemiresistive variation when exposed to analyte gases that explains the increased gas response to all the gases. Notably, the basic properties of MgO enhanced the selectivity to C_2_H_5_OH. However, the use of acid–base properties to enhance response and selectivity to C_2_H_5_OH may hamper the selective detection of other gases aside from C_2_H_5_OH.

### 3.3. Core-Shell Structures

Among various heterostructures based on highly crystalline metal oxide NWs, and aside from the discrete decoration of nanoparticles, the complete coverage of a shell layer on an NW has also been strongly investigated in the field of nanoelectronics, including gas sensors. Due to their functional properties arising from interconnected junctions between different materials, coaxial nanocables with core–shell structures can exhibit superior gas-sensing characteristics compared to pristine NW-based gas sensors. The conduction across *p*(core)-*n*(shell) and *n*(core)-*p*(shell) interfaces is significantly different from that within pure *p*-type or *n*-type oxide semiconductors. Moreover, *n*-*n* heterojunctions can also be used to modulate the conduction across the interface when the work function values of two materials are substantially different from each other. When the conduction across core and shell layers becomes difficult, it will occur along the interconnected shell layers in a coaxial nanocable configuration. This is supported by the *p*-type gas-sensing characteristics of ZnO-Cr_2_O_3_ and ZnO-ZnMn_2_O_4_ core-shell nanocables (NCs), as shown in Figure 3c,f. However, even in this case, the conduction is significantly influenced by the modulation of the space charge layer near the interface when the shell layer is very thin. Moreover, the relative portion of electron depletion layer or hole accumulation layer near the surface of the shell layer formed by oxygen adsorption will increase with the thinning of the shell thickness. Thus, the combination of two different sensing materials with different work functions and catalytic properties in a core–shell configuration can provide diverse solutions to achieve gas selectivity and enhance gas sensitivity.

**Figure 6:**
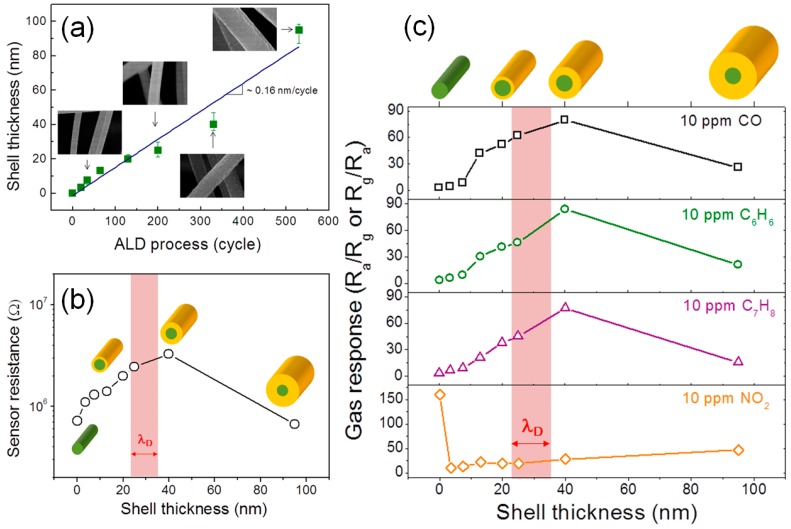
(**a**) Relationship between ZnO shell thickness and number of atomic layer deposition (ALD) cycles; (**b**) sensor resistance in air and (**c**) gas responses of SnO_2_-ZnO core-shell NWs as a function of ZnO shell thickness. Reprinted from [104] with permission. Copyright (2014) American Chemical Society.

Various research groups have reported the synthesis and chemiresistive gas sensor application results of core-shell NWs using *n*-*n* heterojunctions with SnO_2_ and ZnO [101,102,103,104]. Choi et al. [104] prepared various SnO_2_-ZnO core-shell nanocables with different shell thicknesses by controlling the cycle numbers of the atomic layer deposition (ALD) (Figure 6a) and investigated their gas-sensing characteristics. The gas response and sensor resistance in air increased with the increase of the shell layer thickness up to 40 nm and then decreased with further thickening (Figure 6b,c). The decrease of sensor resistance in air and gas response with increasing shell thickness from 40 to 95 nm can be attributed to the increase of the nondepleted region in the shell layers, which explains that the core-shell configuration is effective to enhance gas response. However, the decrease of sensor resistance with decreasing shell layer thickness from 40 to 3 nm cannot be explained if it is assumed that the conduction occurs only along the shell layers. Accordingly, Choi et al. attributed this decrease of sensor resistance by conduction both along the shell layer and near-interface core layers to the “electric field smearing effect”.

The formation of core-shell structures by thermal evaporation of chloride-based source powders of shell layers next to the growth of core oxide NWs has been reported by tuning the reaction temperature and time [80,82,83,84]. An increase in temperature, time, or both, initially resulted in the abundant formation of nanoparticles on the surface of the NWs. The continuous reaction causes the necking of the nanoparticles, ultimately leading to complete coverage. Various shell layers of *p*-type oxide semiconductors (Cr_2_O_3_, ZnMn_2_O_4_, Co_3_O_4_) have been coated on ZnO and SnO_2_ NWs to form core-shell structures [80,81,84,105]. However, due to the conduction through *p*-type oxide semiconductors with relatively low gas response and quite thick shell layer with small contribution of modulating the space charge layer near the interface, the gas response was not high (Figure 3c,f).

Ga_2_O_3_-based gas sensors reported in the literature show efficient gas sensing results of O_2_, H_2_, CO, and CH_4_ gases [106,107,108,109,110]. However, most results using Ga_2_O_3_ as sensing material require very high operating temperatures in the range 600–1000 °C. The deposition of a ZnO or SnO_2_ shell layer using ALD on the surface of CVD-grown Ga_2_O_3_ NWs has proven effective not only for enhancing the selectivity, but also decreasing the sensing temperature by lowering the sensor resistance [111,112].

Since the gas-sensing reaction (using metal oxide semiconductor-based gas sensors) mainly occurs on the surface of the nanostructure, the selective detection of a specific gas is rather challenging using core-shell NWs. However, highly sensitive core-shell NWs can be a good platform to achieve gas selectivity because less reactive gases can be detected by the combination of highly sensitive sensing materials with catalytic filtering overlayers. Further studies are required for this in connection with the development of synthetic processes for core-shell NWs.

### 3.4. New Physicochemical Route for the Preparation of p-Type Metal Oxide Nanowires

Gas responses of oxide semiconductors are determined by the charge carrier concentration (receptor function), conduction across nanostructures (transducer function), and gas accessibility to the sensing surfaces (utility factor) [113]. In the sole viewpoint of transducer function, the gas responses of *p*-type metal oxide semiconductors such as NiO, CuO, Cr_2_O_3_, and Co_3_O_4_ are known to be lower compared to those of *n*-type metal oxide semiconductors such as In_2_O_3_, SnO_2_, and ZnO, assuming they possess the same morphological configuration [114]. Therefore, the application of resistive type gas sensors using *p*-type semiconductors is still in the initial investigation stage. However, *p*-type metal oxide semiconductors show excellent catalytic properties to oxidize various volatile organic compounds due to their abundant oxygen adsorption [115] and multivalent characteristics. In this respect, *p*-type metal oxide semiconductors are considered as promising sensing materials for detecting various volatile organic compounds [77].

Many studies have been performed on the preparation of nanorods [116,117,118], hollow spheres [119,120], hierarchical nanostructures [121,122,123], and monolayer inverse opals [124] of *p*-type oxide semiconductors by solution-based routes. In contrast, the growth of *p*-type oxide semiconductor NWs has been barely investigated. Although the synthesis of CuO NWs by thermal oxidation of Cu has been reported [23,27,28], most *p*-type metal oxide semiconductors—such as Co_3_O_4_, Cr_2_O_3_, NiO, and Mn_3_O_4_—are difficult to grow using the thermal evaporation method due to their low vapor pressure and high melting point of the source powders.

Recently, the successful transformation of template NWs into the desired compound materials has been reported, and has proven promising as a method to prepare NWs that are difficult to synthesize using conventional routes. Methods such as alloy formation, galvanic replacement, and cationic and anionic exchange have been researched as means of achieving this transformation [125]. In the case of vapor-phase transformation, cationic and anionic transformations are the most promising methods, and have been successfully demonstrated in transforming Ga_2_O_3_ NWs into GaN NWs, ZnO NWs into ZnS NWs, CdS NWs into ZnS NWs, CdS NWs into Cu_2_S NWs, etc. [126,127,128,129,130]. In these cases, gases such as NH_3_ and H_2_S are used for anionic exchange, while metal chlorides are used for the substitution of metal cations. However, the transformation of *n*-type into *p*-type metal oxide NWs maintaining high crystallinity by vapor-phase method have not been reported until recent years.

**Figure 7:**
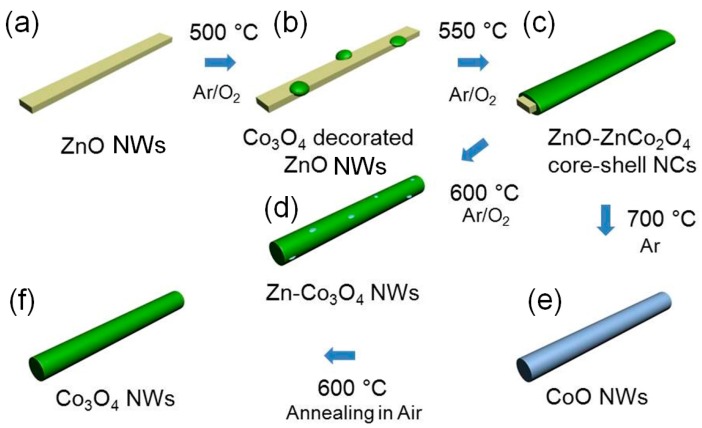
A schematic illustration of the transformation from ZnO NW to CoO NW and Co_3_O_4_ NW. Adapted from [105] with permission. Copyright (2012) The Royal Society of Chemistry.

The present authors [105] have successfully transformed single crystalline ZnO NWs into various *p*-type semiconductor NWs by the vapor-phase method. ZnO NWs prepared by the thermal evaporation method was transformed into Co_3_O_4_-containing NWs by using CoCl_2_ powder as a cation source at 500–700 °C (Figure 7). When the reaction between ZnO NWs (Figure 7a) and CoCl_2_ was carried out at 500 °C, the surface of the ZnO NWs were decorated by Co_3_O_4_ nanoparticles (Figure 7b). At 550 °C, ZnO-ZnCo_2_O_4_ core-shell nanocables were obtained (Figure 7c), while at 600 °C, Co_3_O_4_ NWs’ trace concentration of residual Zn remained (Figure 7d). When the temperature was raised to 700 °C, complete transformation of ZnO NWs into CoO NWs was successful (Figure 7e), and by annealing CoO NWs at 600 °C, phase-pure Co_3_O_4_ NWs could be obtained (Figure 7f) [105]. Similarly, full conversion of ZnO NWs into Mn_3_O_4_ NWs and NiO NWs was possible by thermal evaporation of MnCl_2_ and NiCl_2_ at 750 and 700 °C, respectively [84,131].

**Figure 8:**
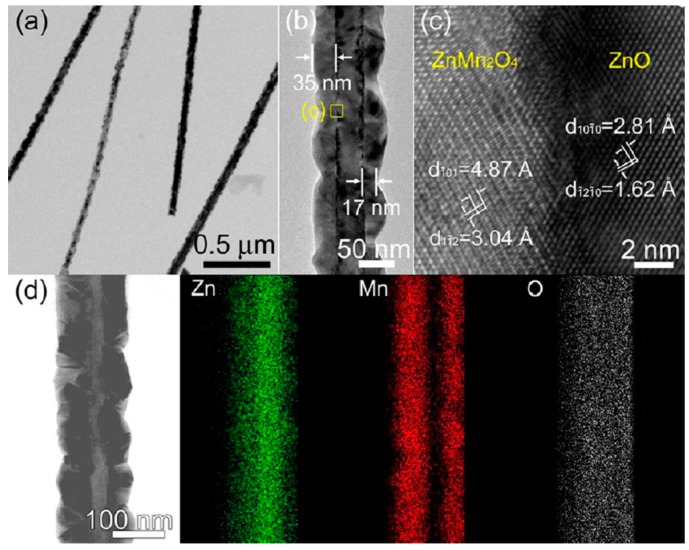
Morphologies and crystal structures of ZnO-ZnMn_2_O_4_ NCs: (**a**,**b**) TEM images of ZnO-ZnMn_2_O_4_ NCs grown on Si substrates; (**c**) Lattice-resolved image of ZnO-ZnMn_2_O_4_ NCs; (**d**) Energy dispersive X-ray spectroscopy (EDS) elemental mapping of Zn, Mn, and O. Reprinted from [84] with permission. Copyright (2012) American Chemical Society.

The authors [84] proposed possible transformation reaction mechanisms on the basis of cation exchange reactions reported in the literature. In the case of the transformation of ZnO NWs into Mn_3_O_4_, the initial formation of the intermediate ZnO-ZnMn_2_O_4_ core-shell structure at 600 °C (Figure 8) is explained by the following reaction:

3ZnO(s) + 2MnCl_2_(g) + 1/2O_2_(g) → ZnMn_2_O_4_(s) + 2ZnCl_2_(g) (1)

**Figure 9:**
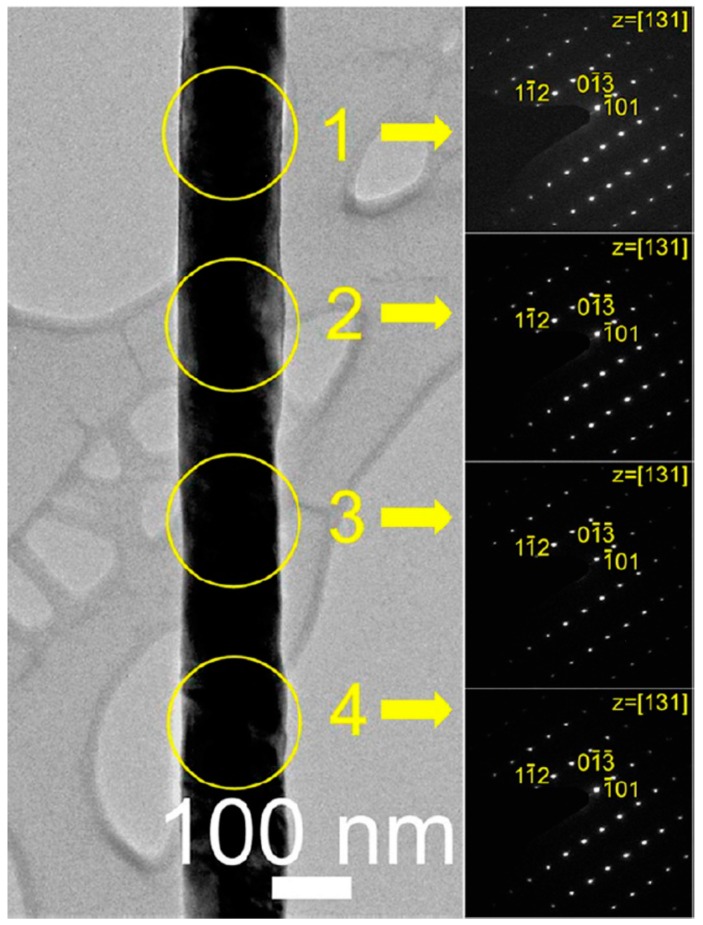
TEM image and selected area electron diffraction (SAED) patterns for the Mn_3_O_4_ NW. Reprinted from [84] with permission. Copyright (2012) American Chemical Society.

This reaction is possible due to the comparably low melting point of the MnCl_2_ powder (652 °C). By increasing the reaction temperature, further conversion of ZnMn_2_O_4_ into Mn_3_O_4_ occurs under very high MnCl_2_ vapor pressure and consequently enhances the cation exchange reaction at 750 °C (Figure 9), according to the following reaction:

ZnMn_2_O_4_(s) + MnCl_2_(g) → Mn_3_O_4_(s) + ZnCl_2_(g) (2)

The selected area electron diffraction patterns along with the longitudinal direction of the NWs (Figure 9) confirm the preparation of single crystalline Mn_3_O_4_ NWs. This is explained by the heteroepitaxial growth of tetragonal spinels on ZnO NWs and suggests that the single crystalline *p*-type oxide NWs can be prepared when the crystal structures and ion sizes of two different oxide NWs (before and after exchange reaction) match well. The overall transformation reaction of ZnO NWs into *p*-type metal oxide NWs greatly depends upon the vapor pressure of metal chloride sources, and this should be carefully controlled by tuning the reaction temperature. This vapor-phase method is a promising route to prepare highly crystalline *p*-type metal oxide NWs as well as different configurations of *p*-*n* heterostructures such as decorated nanostructures and core-shell nanocables by finely tuning the reaction conditions.

### 3.5. Hierarchical Structures

The basic principle behind resistive gas sensors is the surface reaction between adsorbed oxygen atoms and analyte gas molecules. Therefore, increasing the surface area and number of chemiresistive contacts of the nanostructures has always been the main strategy for the enhancement of the gas sensing characteristics. Since the formation of brush-like hierarchical structures can increase not only the surface area but also the number of NW-to-NW contacts, it can be considered as a useful approach in order to enhance the sensing properties. This is feasible when considering the potential barriers formed at the NW-to-NW contacts, as supported by a report on the increase of gas response with increasing number density of NWs in NW network sensors [12].

Brush-like hierarchical NWs were initially synthesized using a single material such as ZnO and SnO_2_ [132,133]. For the growth of branches on backbone NWs, methods such as hydrothermal and thermal evaporation can be used during the second step of the reaction. Heteronucleation during the hydrothermal process results in the formation of nanorods on the NWs’ surfaces, as usually the interfacial energy between crystal nuclei and substrates is smaller than that between crystal nuclei and solutions. The synthesis of hierarchical structures using the thermal evaporation method usually requires two different source powders for backbone and branches. The growth of backbone NWs is the result of the VLS mechanism, while the branches are formed by the vapor-solid (VS) mechanism. The growth of branches by the VS method without a growth catalyst is possible through the formation of new growth sites following the adsorption of metal vapor on the surface of metal oxide NWs that become nucleation sites. Hierarchical NWs show enhanced gas-sensing characteristics compared to pristine NWs due to their higher surface area providing more active sites, higher porosity arising from their network structure, and branch-to-branch contact points creating additional potential junctions for larger resistance variation.

Aiming at further improving the gas-sensing characteristics of hierarchical structures using metal oxide NWs, many research groups proposed to combine different materials in order to obtain an additional catalytic effect. Branched NWs using a combination of materials among SnO_2_, ZnO, and W_18_O_49_ have been reported [134,135,136]. By the formation of branches on backbone NWs using the aforementioned materials, *n*-*n* heterojunctions are formed at the branch/backbone and branch/branch interfaces. Although the abundant potential junctions cause an enhancement of gas response, the combination of two different *n*-type metal oxide semiconductors still shows selectivity limited to C_2_H_5_OH gas.

For the selective detection of other toxic gases, the use of suitable catalytic materials is necessary. It is generally difficult to detect aromatic hydrocarbons such as benzene, toluene, and xylene (BTX) with relatively low reactivity using pristine *n*-type oxide semiconductor NWs. The *p*-type metal oxide semiconductors, in contrast, are known to be excellent catalysts to promote the oxidation of comparably less reactive BTX gases owing to their multivalence properties and abundant oxygen adsorption. In this perspective, the doping or surface modification of hierarchical NWs using such catalytic materials could be an effective approach to selectively detect other harmful gases.

**Figure 10:**
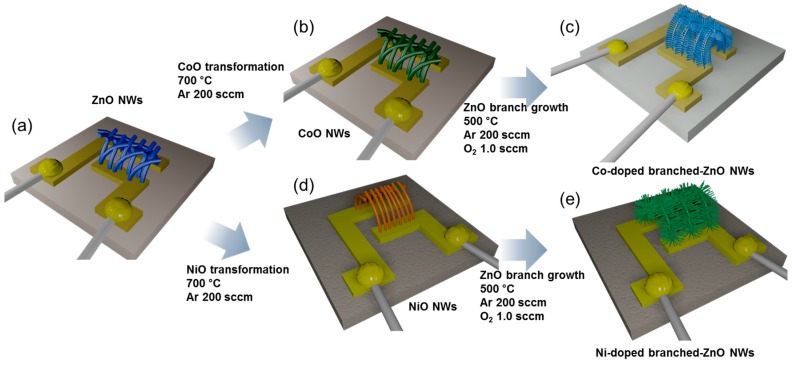
(**a**) Growth of ZnO NWs on alumina substrate with Au electrodes; (**b**) transformation of ZnO NWs into CoO NWs; (**c**) growth of Co-doped branched ZnO NWs from CoO NWs; (**d**) transformation of ZnO NWs into NiO NWs; (**e**) growth of Ni-doped ZnO NWs from NiO NWs. Reproduced from [131,137] with permission; (**a**–**c**) [137] Copyright (2014) American Chemical Society; (**d**,**e**) [131] Copyright (2015) Elsevier.

**Figure 11:**
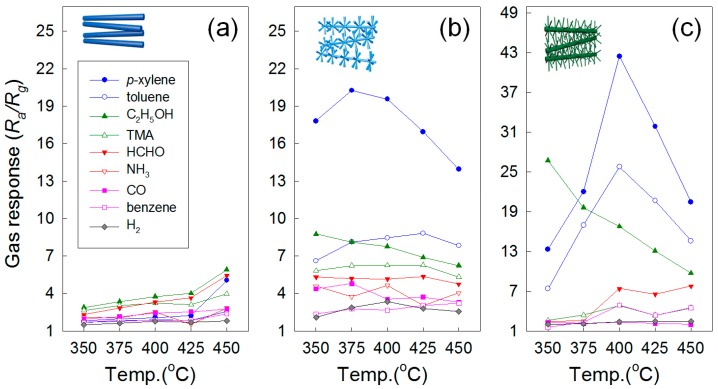
Gas response of (**a**) pristine ZnO; (**b**) Co-doped branched ZnO NWs; and (**c**) Ni-doped branched ZnO NWs to 5 ppm *p*-xylene, toluene, C_2_H_5_OH, TMA, HCHO, NH_3_, CO, benzene, and H_2_ at 350–450 °C. Reproduced from [131,137] with permission; (**a**,**b**) [137] Copyright (2014) American Chemical Society; (**c**) [131] Copyright (2015) Elsevier.

The research group of the present authors proposed a unique method for the synthesis of *p*-type metal oxide NWs by transformation of ZnO NWs, and advanced the method for the formation of catalyst-doped hierarchical NWs (Figure 10). ZnO NWs (Figure 10a) were successfully transformed into *p*-type CoO (Figure 10b) and NiO NWs (Figure 10d) via thermal evaporation of metal chloride powders by the cationic exchange reaction [131,137]. ZnO branches were grown on the surface of CoO and NiO NWs by thermal evaporation using Zn metal powder (Figure 10c,e). A small amount of core CoO and NiO remained after the growth of branched ZnO NWs; thus, ZnO hierarchical NWs uniformly doped with Co or Ni were prepared. In the process of branch growth, the *p*-type metal oxides acted as catalysts in the VLS mechanism, and by fine tuning the reaction temperature, residual transition metals remained within the lattice as dopants (Figure 10). Both Co- and Ni-doped branched ZnO NW network gas sensors (Figure 10c,e) showed highly selective and sensitive detection of xylene gas, while pure ZnO NW network gas sensors showed high response to C_2_H_5_OH (Figure 11). The enhanced gas response and selectivity to less reactive BTX gases were attributed to the catalytic activity of Co- and Ni-dopants and increased potential junctions at the branch/branch contacts.

This novel method of spontaneous doping of catalytic materials in hierarchical NWs was never reported before. Aside from the use of CoO and NiO as growth catalysts and templates for the synthesis of hierarchical structures, appropriate *p*-type metal oxides or catalytic materials could be used for the functionalization of metal oxide NW-based gas sensors to enhance their selectivity to various gases. Although further studies are required for the successful transformation and spontaneous doping of catalytic materials using other metal oxides, nonetheless, this method has paved a new way for achieving selective detection of various harmful gases using oxide NW network sensors. If such sensors were to be successfully fabricated, ultimately, they could be used in a sensor array for e-nose to recognize complex chemical quantities in various applications.

### 3.6. Oxide Nanowires for Selective Gas Detection

**Figure 12:**
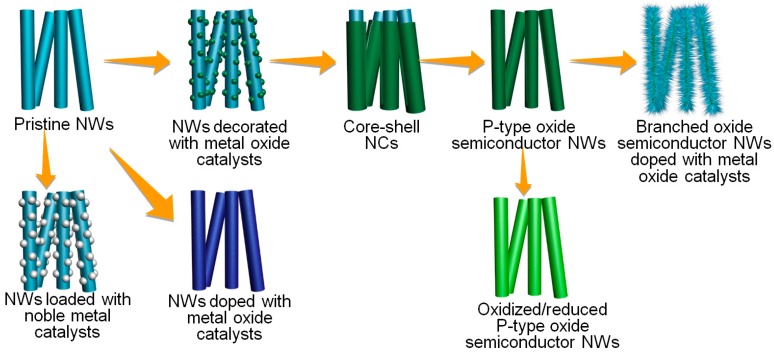
Various oxide nanowires for highly selective gas detection.

Physicochemical modification of oxide NWs provides diverse strategies to achieve selective gas detection. Schematic structures of various oxide NWs reviewed in this contribution were summarized in Figure 12. The oxide semiconductor NWs can be decorated with metal oxide catalysts as well as noble metal catalysts to promote the sensing of a specific gas. The doping of catalytic materials within the lattice of sensing materials may be considered when the ionic sizes of dopant and host cation are similar to each other. The *n*-type oxide NWs can be transformed either to core(*p*-type oxide semiconductor)-shell(*n*-type oxide semiconductor) NCs or to *p*-type oxide semiconductor NWs through the control of reaction temperature and time in vapor-phase cation exchange reaction, both of which can be used to enhance or tune the gas selectivity. Finally, the catalyst-doped branched oxide NWs exhibit superior gas selectivity by catalytic promotion of a specific gas-sensing reaction as well as high gas response by the increase of chemiresistive branch-to-branch junctions.

## 4. Other Approaches to Enhance Gas Selectivity

Molecular sieving can be used to enhance gas selectivity of NW-based gas sensors. Drobek et al. [138] reported that the encapsulation of ZnO NW sensors with MOF (metal organic frameworks) enhance the selectivity to hydrogen. The molecular sieving of large molecules such as toluene and benzene was suggested as the reason for gas selectivity.

The selectivity to a specific gas can be enhanced by the optimized design of sensing materials, catalysts, and hetero-nanostructures. However, it is still challenging to detect huge numbers of different analyte gases in a selective manner because of the simple sensing principle of oxide chemiresistors or chemical similarity between analyte gases. When highly selective gas detection is difficult, pattern recognition of analyte gas using the array of NW sensors can be a good alternative for gas identification [139,140]. For instance, Baik et al. [61] made an electronic nose using the array of pure, Pd-, and Ag-loaded SnO_2_ NW sensors, and demonstrated the discrimination between ethylene, CO, and H_2_. Sysoev et al. [12] fabricated the sensor array by coating different number density of SnO_2_ NWs to multielectrodes and reported that air, CO, ethanol, and 2-propanol can be discriminated successfully by the linear discriminant analysis of sensor signal. They further showed that the discrimination power is reinforced when the temperature gradient is additionally applied to the sensor array. Note that the array of oxide NW sensors can be integrated into a small microchip for artificial olfaction with low power consumption, which can open new applications for mobile chemical sensors with the progress of Internet of Thing (IoT) and sensor network. For this, a wide range of oxide-based NW sensors with different gas-sensing characteristics are essential.

## 5. Conclusions

Single crystalline metal oxide NWs grown using the CVD method have attracted much attention in the past years for their unique properties that make them valuable for numerous applications. Due to their large surface-to-volume ratio, high thermal stability, and porous network structure combined with a low tendency to form aggregates, the use of metal oxide semiconductor NWs in the field of chemical sensors has been extensively studied. Over the years, *n*-type metal oxide semiconductor NWs such as SnO_2_, ZnO, and In_2_O_3_ have been applied as resistive-type gas sensors, showing high and fast responses. However, the selective gas detection using oxide NWs has always been a challenging issue. In this review, the authors have summarized and proposed various early and recent strategies to enhance the gas selectivity of NW network-based gas sensors. These include doping and loading of noble metals, doping and decorating of catalytic metal oxides, formation of core-shell structures, transformation of *n*-type NWs into *p*-type NWs, and design of hierarchical NWs. Many reports in the literature have provided encouraging results, however, most examples have shown selectivity to C_2_H_5_OH and NO_2_, which are two of the most common target gases in the field of gas sensors. The authors wish to stress the importance of the use of *p*-type metal oxides as catalysts and sensing materials for the selective detection of other harmful gases, and the methods to either dope or decorate the catalytic materials in backbone NWs. Nevertheless, continuous studies are required in order to discover suitable catalytic materials for specific gases and new strategies to further refine the selectivity of NWs network based gas sensors.

## Figures and Tables

**Table 1:** Gas sensors using noble-metal-catalysts-loaded nanowires (NWs) in the literature [54,55,56,57,58,59,60,61,62,63,64,65,66,67,68].

Catalyst	Structure	NW Material	Target Gas	References
Pt	Loading	SnO_2_	NO_2_, Toluene, H_2_	[54,55,56]
		ZnO	C_2_H_5_OH	[57]
		In_2_O_3_	O_2_, H_2_	[58,59]
Pd	Loading	SnO_2_, VO_2_	H_2_	[60,61,62]
Au	Loading	ZnO, In_2_O_3_	CO, C_2_H_5_OH	[59,63,64,65]
		ZnO	C_2_H_5_OH	
Ag	Loading	SnO_2_	C_2_H_5_OH	[66,67,68]

**Table 2:** Metal-oxide-catalyst-doped and -decorated NWs and their target gases [78,79,80,81,82,83,84,85,86,87,88,89,90].

Catalyst	Structure	NW Material	Target Gas	References
CuO	Decoration	SnO_2_	H_2_S	[78,79]
Cr_2_O_3_	Decoration	ZnO, SnO_2_	TMA	[80,81]
Co_3_O_4_	Decoration	ZnO	NO_2_, C_2_H_5_OH	[82]
NiO	Decoration	ZnO	C_2_H_5_OH, HCHO	[83]
Mn_3_O_4_	Decoration	ZnO	C_2_H_5_OH	[84]
La_2_O_3_	Decoration	SnO_2_	C_2_H_5_OH	[85]
Cu, CuO	Doping	SnO_2_	H_2_S	[86,87]
Mo, Mo_3_O_4_	Doping	ZnO	H_2_S	[88]
Mg, MgO	Doping	ZnO	C_2_H_5_OH	[89]
Sb	Doping	SnO_2_	C_2_H_5_OH	[90]
